# Supplementary material for: Gut microbiome shifts in adolescents after sleeve gastrectomy with increased oral-associated taxa and pro-inflammatory potential
Source: medRxiv. 2024 Sep 16:2024.09.16.24313738. Preprint. [Version 1] doi: 10.1101/2024.09.16.24313738 (PMC11451705; doi:10.1101/2024.09.16.24313738)
Supplement: Supplement 3 — (A) Identified viruses were primarily comprised of Caudoviricetes. Donut plot represents abundances across both pre-VSG (left) and post-VSG (right) samples for each taxonomic category. (B) Host taxonomy of identified viruses including Bacteroides, Bacteroidaceae, and Prevotella, as well as several with no known hosts. As in (A), plot represents summarized abundances across both pre-VSG (left) and post-VSG (right) samples for each viral host. (C) Viral taxonomic diversity does not change due to VSG with respect to either Chao1 richness or evenness. (D) Viral taxonomic composition does not change signficantly due to VSG. Exploration of either (E) viral Protein Family (PFAM) diversity or (F) composition did not reveal significant differences due to VSG. Points indicate individual samples and lines connect paired samples. P-values for diversity were generated from a linear mixed-effects model with the subject as the random effect. Ellipses in composition plots represent 20% confidence intervals for each group. PERMANOVA calculated the R2 and p-values with the subject as the strata variable. Abbreviations: VSG: vertical sleeve gastrectomy, PERMANOVA (Permutational Analysis of Variance), PFAM (Protein Family). [file media-3.pdf]

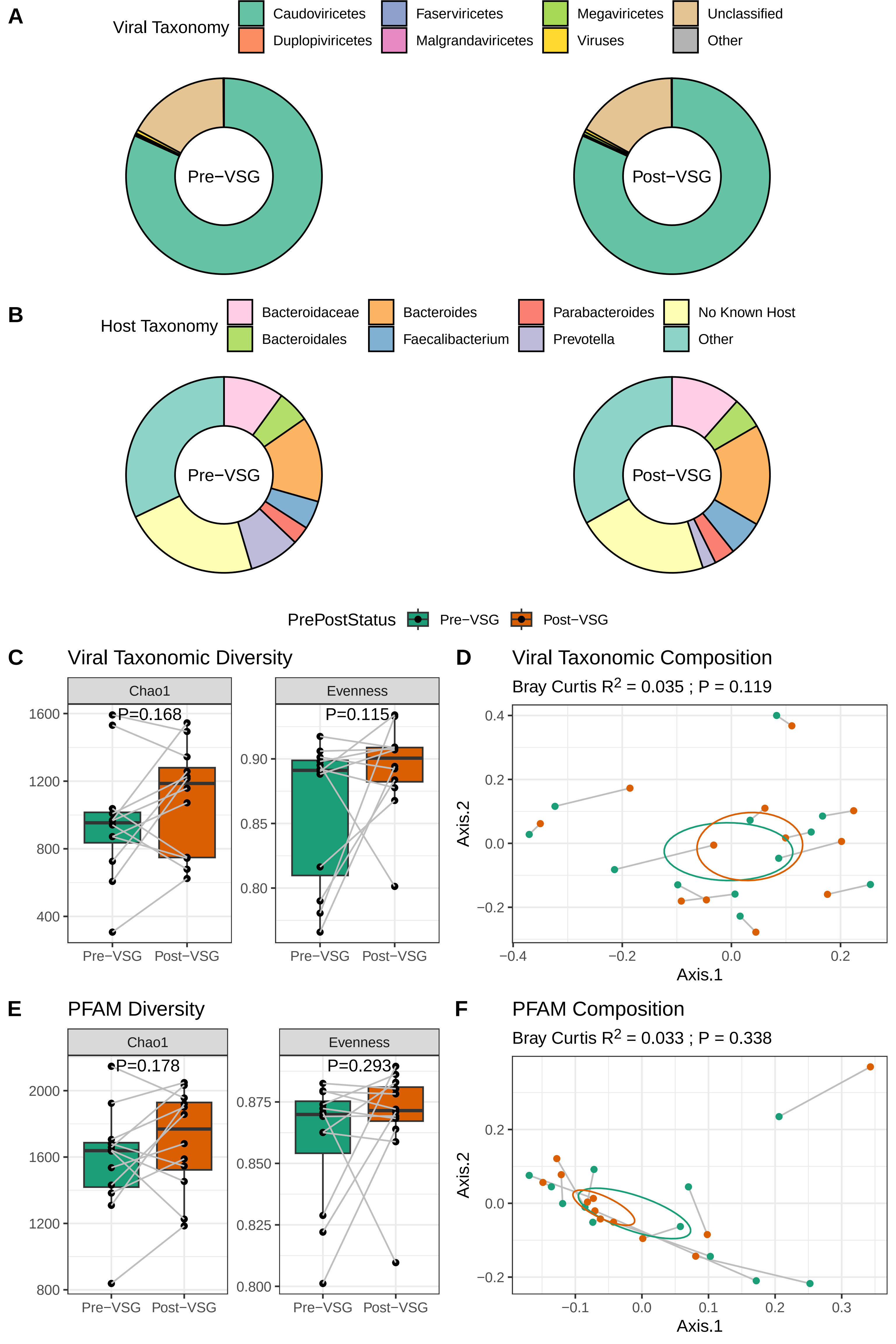

A
Viral Taxonomy
Caudoviricetes
Duplopiviricetes
Faserviricetes
Malgrandaviricetes
Megaviricetes
Viruses
Unclassified
Other
Pre−VSG
Post−VSG
B
Host Taxonomy
Bacteroidaceae
Bacteroidales
Bacteroides
Faecalibacterium
Parabacteroides
Prevotella
No Known Host
Other
Pre−VSG
Post−VSG
PrePostStatus
Pre−VSG
Post−VSG
C
Viral Taxonomic Diversity
Chao1
P=0.168
Evenness
P=0.115
D
Viral Taxonomic Composition
Bray Curtis R2 = 0.035 ; P = 0.119
Axis.1
Axis.2
E
PFAM Diversity
Chao1
P=0.178
Evenness
P=0.293
F
PFAM Composition
Bray Curtis R2 = 0.033 ; P = 0.338
Axis.1
Axis.2
